# Posterior Shoulder Pain and Muscle Wasting in an Older Adult

**DOI:** 10.7759/cureus.28850

**Published:** 2022-09-06

**Authors:** Naomi Kelley, Morteza Khodaee

**Affiliations:** 1 Orthopedics, University of Colorado School of Medicine, Aurora, USA; 2 Family Medicine & Orthopedics, University of Colorado School of Medicine, Aurora, USA

**Keywords:** muscular atrophy, shoulder injuries, rotator cuff tear management, rotator cuff tears, rotator cuff arthropathy

## Abstract

Musculoskeletal injuries are among the most common chief complaints in the geriatric population. Shoulder pain with associated deformity should be evaluated for possible joint dislocations, fractures, and musculotendinous tears. A comprehensive evaluation beginning with history and physical examination is important. Typical imaging utilized for the diagnosis of shoulder injuries includes plain radiography, ultrasound, and magnetic resonance imaging (MRI). We present a case of a 75-year-old male with massive rotator cuff tears and subsequent shoulder deformity. Management with non-surgical or surgical approaches should begin as soon as possible to delay the development of rotator cuff arthropathy.

## Introduction

Pain and deformity of the shoulder secondary to an acute injury is relatively common among elderly patients. A thorough history and physical examination is essential to narrow down the broad potential differential diagnosis of shoulder injuries. Acutely, shoulder deformity may indicate a fracture, dislocation, or tendon rupture. A fracture nonunion, mal-reduced dislocation, or neuromuscular injury should be considered if a deformity persists several months after the initial injury [1,2]. Furthermore, significantly limited range of motion (ROM) is concerning for a musculotendinous injury or chronically dislocated or subluxed glenohumeral joint. Here, we present and discuss the appropriate evaluation of an elderly patient with a chronic massive rotator cuff tear.

## Case presentation

**Figure 1:**
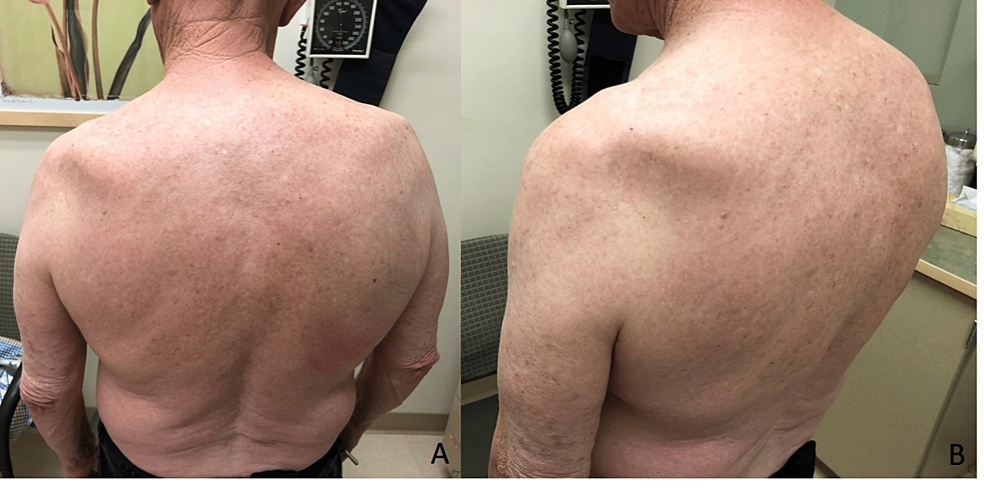
Abnormal left posterior shoulder contour with wasting below the lateral aspect of the spine of the scapula (A, B).

A 75-year-old male presented with a two-month history of left shoulder pain after attempting to lift a washing machine. Upon injury, he described feeling a painful pop in the back of his shoulder. The pain was localized to the posterior aspect of his left shoulder. It was constant and progressively worsened throughout the day. He denied numbness and tingling in his left upper extremity as well as instability of the shoulder. He used ice, heat, rest, and ibuprofen intermittently without significant relief. Physical examination revealed significant atrophy in the left shoulder just below the scapular spine (Figure 1A-1B).

**Figure 2:**
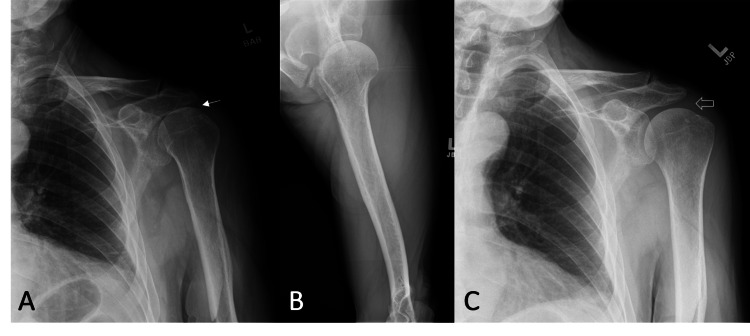
Left anteroposterior (A) and axillary (B) views revealed mild proximal humeral head migration (arrow) with mild glenohumeral and moderate acromioclavicular joints degenerative joint disease. Proximal humeral head migration (open arrow), a radiographic sign of rotator cuff arthropathy, was not present a year earlier (C).

**Figure 3:**
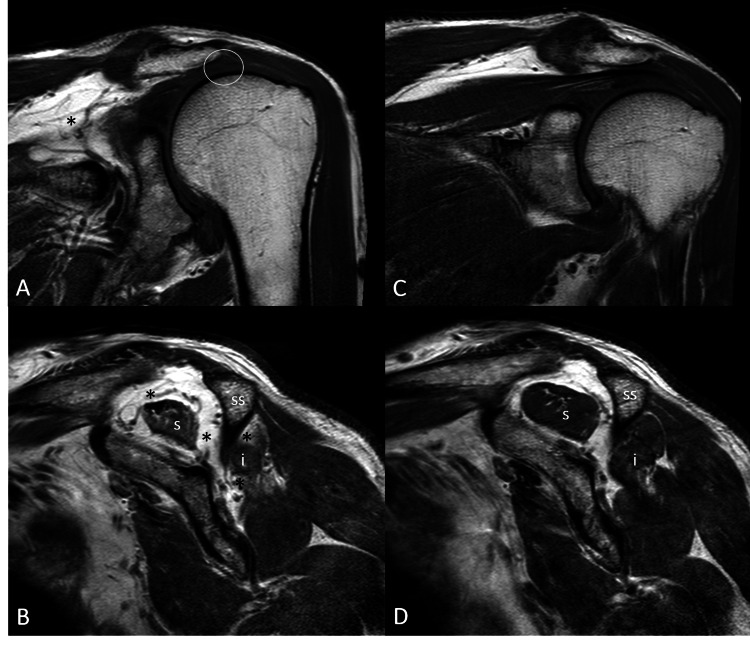
Absence of supraspinatus tendon (circle) on a coronal (A) and sagittal (C) T1-weighted image. This is due to the massive rotator cuff tear involving nearly the entirety of the supraspinatus (s) and infraspinatus (i) tendons. Both muscle bellies demonstrate progressive severe fatty atrophy (*) which was not present in a previous MRI a year earlier (C and D). ss: scapular spine

He had symmetrical shoulder ROM with forward flexion to 160°, abduction to 160°, external rotation to 70°, and internal rotation to reach T7. He had 3/5 left external rotation strength but otherwise normal shoulder strength. Bilateral empty can, drop arm, lift off, Hornblower, Neer, and Hawkins impingement tests were negative. There was no evidence of scapulothoracic dyskinesis. All neurovasculature was intact. Plain radiography revealed proximal humeral head migration (Figure 2A-2C) and MRI showed massive supraspinatus and infraspinatus tendon ruptures with fatty atrophy that were not seen on prior imaging (Figure 3A-3D).

**Figure 4:**
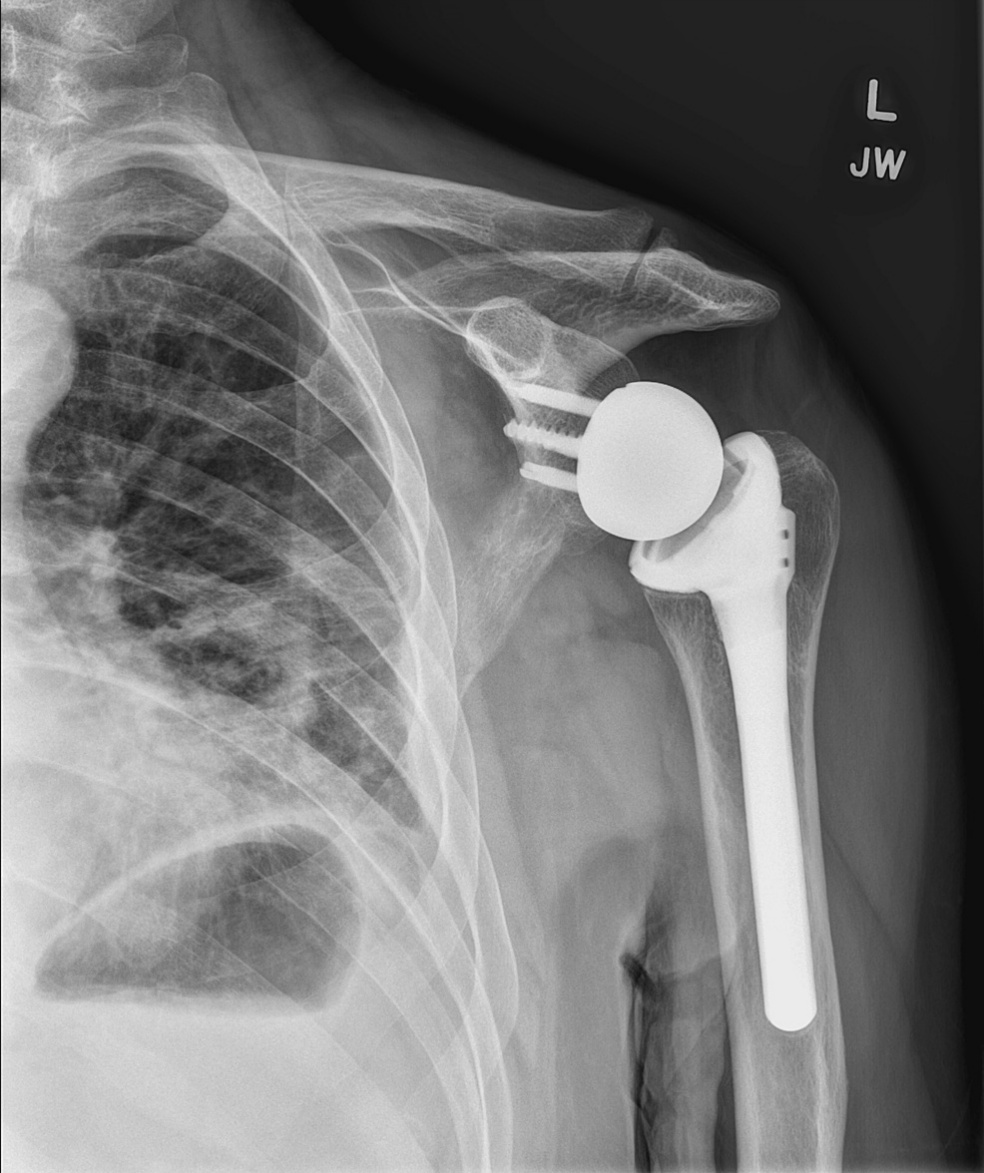
Well-aligned left reverse total shoulder arthroplasty with no evidence of implant failure or migration two years post-operatively.

The final diagnosis was a left massive rotator cuff rupture including infraspinatus and supraspinatus tendons. Per Orthopedic Surgery, surgical repair of the ruptured tendons was not recommended due to the patient’s age, degree of atrophy, and chronicity of the tear. Instead, the patient participated in physical therapy consisting of strengthening and ROM programs for eight months. Unfortunately, due to persistent functional impairment and persistent pain, the patient elected to proceed with a left reverse total shoulder arthroplasty 10 months after the initial injury. The patient recovered well with physical therapy with restored strength and painless shoulder ROM two years post-operatively (Figure 4).

## Discussion

When evaluating musculoskeletal injuries, there are several broad injury categories that should be considered. These conditions include but are not limited to fractures, dislocations, neurologic and soft tissue injuries. Scapular fractures and posterior shoulder dislocations should be considered in the setting of an acute injury with subsequent shoulder pain and asymmetric posterior deformity. However, fractures and dislocations are less likely given the chronicity of the injury, the patient’s relative normal ROM, lack of swelling or hematoma formation, and unremarkable plain radiography [1]. A neurologic injury such as cervical plexopathy may cause asymmetric atrophy, but would likely exhibit radiating, burning pain that is exacerbated by neck ROM. Although relatively rare, suprascapular neuropathy can also result in rotator cuff (specifically supraspinatus and/or infraspinatus muscles) atrophy and functional impairment but would likely be aggravated by shoulder adduction and internal rotation [2]. Muscular atrophy due to suprascapular neuropathy is usually evident throughout the entire supraspinatus and/or infraspinatus muscle. In contrast, our patient’s deformity was localized to the lateral aspect of the shoulder as a result of muscle retraction.

Given that rotator cuff tears (RCTs) are present in nearly half of adults over 70 years of age [3], one must have a high index of suspicion for these injuries when evaluating shoulder pain and dysfunction in an elderly patient. RCTs can be categorized based on the location, length and depth of the tear as well as the degree of tendon retraction [4]. There is no universally accepted definition for a massive rotator cuff tear [5,6]. It has been defined as a tear with a diameter ≥5 cm or complete detachment of ≥2 rotator cuff tendons. Massive RCTs have been reported to have a prevalence of about 20% of all RCTs [5-7]. In this case, the patient’s posterior shoulder pain, external rotation weakness, and asymmetric left shoulder atrophy are most suggestive of a chronic massive infraspinatus tendon tear. The patient’s otherwise normal strength examination with other special tests suggests that the injury is less likely to involve other rotator cuff muscles. Of note, normal shoulder abduction cannot rule out a massive supraspinatus tear due to compensation by the deltoid muscle as seen in this patient [8].

RCTs classically present with localized pain and/or weakness associated with each rotator cuff muscle while performing several well-described special tests. For example, an infraspinatus or teres minor tendon tear will exhibit pain and weakness with external rotation. Specifically, a positive external rotation lag sign is highly suggestive of an infraspinatus tear whereas the teres minor will have a weakness with the Hornblower’s test [9]. A subscapularis tear is best evaluated with the lift-off test [9]. Lastly, a supraspinatus tear is commonly evaluated with the empty can test and drop arm sign. All of these special tests have varied sensitivities and specificities and should be used in conjunction with clinical judgement and advanced imaging [9]. Despite commonly presenting as shoulder pain, RCTs are asymptomatic in more than 65% of adults [10]. The exact prevalence of asymptomatic massive RCTs is unknown [11].

A plain radiography should be obtained to evaluate for a fracture or dislocation. However, more advanced imaging will be required for soft tissue injuries. Ultrasound is a valuable modality, specifically as a bedside diagnostic tool. MRI is the best imaging to evaluate both the severity, chronicity, and pattern of RCTs [4].

In the case of symptomatic RCTs, there are several nonoperative and operative treatment options available. Treatment should be started as soon as possible to preserve shoulder function (e.g., ROM, strength) and prevent fatty degeneration and arthropathy [5-7, 11-14]. Unfortunately, there is no high-quality randomized controlled trial comparing different nonoperative and operative management, specifically for massive RCTs [5-7, 12]. The choice of treatment is contingent upon patient demographics, RCT features (location, size, tendon involvement, chronicity) [4], comorbidities, as well as the presence of glenohumeral osteoarthritis (rotator cuff arthropathy) among other associated shoulder pathologies [5,6,11,12]. Nonoperative management includes analgesics, physical therapy (focusing on improvement of ROM, strength, and function), and subacromial space corticosteroid injections (mainly for symptom relief) [5-7, 13]. Operative management includes tissue debridement, subacromial decompression, biceps tenotomy, tendon transfers, and tendon repair [5,6,12]. In elderly patients with degenerative glenohumeral joint disease and rotator cuff atrophy, a reverse total shoulder arthroplasty may be indicated [5,6,12]. In this case, an elderly patient with a chronic massive rotator cuff tear was unlikely to benefit from surgical repair. Instead, appropriate initial treatment consisted of targeted physical therapy, subacromial corticosteroid injections, and analgesics [13]. Despite these conservative management, the patient had persistent pain and functional impairment and subsequently received a reverse total shoulder arthroplasty.

## Conclusions

When managing geriatric patients with acute and subacute musculoskeletal injuries with obvious deformity, physicians should be aware of conditions that may cause long-term disability. Evaluation of shoulder pathology includes a thorough history and physical examination with appropriate imaging modalities. Massive rotator cuff tears should be diagnosed and managed as soon as possible to achieve the best patient outcome.
